# High-fat diet-induced hypertension is associated with a proinflammatory T cell profile in male and female Dahl salt-sensitive rats

**DOI:** 10.1152/ajpheart.00389.2018

**Published:** 2018-09-21

**Authors:** Lia E. Taylor, Ellen E. Gillis, Jacqueline B. Musall, Babak Baban, Jennifer C. Sullivan

**Affiliations:** ^1^Department of Physiology, Augusta University, Augusta, Georgia; ^2^Department of Oral Biology, Augusta University, Augusta, Georgia

**Keywords:** aorta, inflammation, kidney, sex differences, T cells

## Abstract

Evidence supports a sex difference in the impact of a high-fat diet (HFD) on cardiovascular outcomes, with male experimental animals exhibiting greater increases in blood pressure (BP) than female experimental animals. The immune system has been implicated in HFD-induced increases in BP, and there is a sex difference in T-cell activation in hypertension. The goal of this study was to determine the impact of HFD on BP and aortic and renal T cell profiles in male and female Dahl salt-sensitive (DSS) rats. We hypothesized that male DSS rats would have greater increases in BP and T cell infiltration in response to a HFD compared with female DSS rats. BP was measured by tail-cuff plethysmography, and aortic and renal T cells were assessed by flow cytometric analysis in male and female DSS rats on a normal-fat diet (NFD) or HFD from 12 to 16 wk of age. Four weeks of HFD increased BP in male and female DSS rats to a similar degree. Increases in BP were accompanied by increased percentages of CD4^+^ T cells and T helper (Th)17 cells in both sexes, although male rats had more proinflammatory T cells. Percentages of renal CD3^+^ and CD4^+^ T cells as well as Th17 cells were increased in both sexes by the HFD, although the increase in CD3^+^ T cells was greater in male rats. HFD also decreased the percentage of aortic and renal regulatory T cells in both sexes, although female rats maintained more regulatory T cells than male rats regardless of diet. In conclusion, both male and female DSS rats exhibit BP sensitivity to a HFD; however, the mechanisms mediating HFD-induced increases in BP may be distinct as male rats exhibit greater increases in the percentage of proinflammatory T cells than female rats.

**NEW & NOTEWORTHY** Our study demonstrates that male and female Dahl salt-sensitive rats exhibit similar increases in blood pressure to a high-fat diet and an increase in aortic and renal T cells. These results are in contrast to studies showing that female rats remain normotensive and/or upregulate regulatory T cells in response to hypertensive stimuli compared with male rats. Our data suggest that a 4-wk high-fat diet has sex-specific effects on the T cell profile in Dahl salt-sensitive rats.

## INTRODUCTION

Two-thirds of hypertension cases in the United States are positively correlated with excessive weight gain (22), and recent data from the National Health and Examination Survey indicate that the prevalence of hypertension increases with body mass index (49). Indeed, in both humans and animal models of hypertension, dietary factors such as saturated fat, cholesterol, and carbohydrates have a profound influence on blood pressure (BP) control (21, 59, 71). There is also evidence supporting a sex difference in the impact of a high-fat diet (HFD) on cardiovascular outcomes (33, 65), with male experimental animals exhibiting greater increases in BP in response to a HFD compared with female experimental animals (19). Although the molecular mechanisms underlying sex differences in the BP response to a HFD are not fully understood, it is now increasingly recognized that T cells and proinflammatory cytokines play an important role in the progression of hypertension in several animal models (4, 15, 20, 29, 39, 41, 42, 67, 69, 76), including Dahl salt-sensitive (DSS) rats (12, 13, 15, 62).

DSS rats, as the name suggests, exhibit progressive increases in BP when switched from a low-salt (0.4%) diet to a high-salt (4%) diet (12). However, several recent studies have shown that male DSS rats also exhibit other forms of diet-induced increases in BP independent of increases in salt intake (47, 48, 62). Most recently, it has been shown that male DSS rats exhibit HFD-induced increases in BP accompanied by increased renal T cell infiltration and injury, which are attenuated after treatment with the lymphocyte inhibitor mycophenolate mofetil (MMF) (62). Furthermore, hypertensive male DSS rats exhibit an increase in aortic inflammation and CD8^+^ T cell infiltration compared with normotensive control rats. Taken together, these findings support a critical role for immune cells in HFD-induced hypertension in male DSS rats; however, female rats were not studied. Based on studies of angiotensin II-induced hypertension (19, 29, 56) and spontaneously hypertensive rats (SHRs) (67, 70) indicating sex differences in T cell activation in hypertension, where hypertensive male animals exhibit a more proinflammatory T cell profile compared with female animals, the present study was designed to test the hypothesis that male DSS rats will have greater increases in BP and T cell infiltration in the vasculature and kidney in response to a HFD compared with female DSS rats.

## METHODS

### 

#### Animals.

Twelve-week-old male and female DSS were used in the present study (Envigo, Prattville, AL). All experiments were conducted in accordance with the National Institutes of Health *Guide for the Care and Use of Laboratory Animals* and were approved and monitored by the Augusta University Institutional Animal Care and Use Committee. Rats were housed in temperature- and humidity-controlled light-cycled quarters and maintained on either a normal-fat diet (NFD; F4031, Bio-Serv, Flemington, NJ) or HFD (F3282, Bio-Serv) from 12 to 16 wk of age (4-wk treatment period). The control NFD consisted of 3.88 kcal/g of gross energy with calories from the following sources: 20.5% protein, 61.6% carbohydrates, and 7.2% fat. The HFD consisted of 5.45 kcal/g of gross energy with calories from the following sources: 20.5% protein, 35.7% carbohydrates, and 36.0% fat. Both diets contain 0.8% NaCl. At the end of all experiments, rats were anesthetized with ketamine-xylazine (50 mg/kg and 6 mg/kg intraperitoneally, respectively, Phoenix Pharmaceuticals, St. Joseph, MO), a thoracotomy was performed, a terminal blood sample was obtained by aortic puncture, and tissues were harvested for flow cytometric analysis of T cells and cytokines.

#### Metabolic parameters.

Body weight, food intake, water intake, and urine output were assessed every 2 wk using metabolic cages in male (*n* = 5) and female (*n* = 6) DSS rats on the NFD or HFD. Systolic BP was assessed in these same rats before and after treatment via tail-cuff plethysmography in rats on a NFD and weekly in rats maintained on a HFD as previously described (55) using an IITC Life Science tail-cuff system (Woodland Hills, CA). Briefly, rats were placed into a rodent restrainer and then into the temperature-controlled warming chamber. The tail-cuff sensor was positioned and secured on the base of the tail, and rats were allowed to acclimate for 10–15 min to the ambient temperature of 32°C before BP recording. Rats were acclimated to the tail-cuff procedure for a minimum of two to three sessions in the days before the first BP readings were recorded, with no more than one BP recording session per day. Reported BP readings are the average of five recordings collected per rat. If a rat appeared in distress, it was removed from the restrainer, and its BP was measured at a later time. One female DSS rat on the NFD died in the restrainer taking final BP measurements. Blood glucose levels were continuously monitored in a subset of these rats on the HFD (*n* = 4 male rats and *n* = 5 female rats) from baseline using HD-XG implantable glucose telemeters (Data Sciences, New Brighton, MN); glucose telemeters were implanted following the manufacturer’s instructions as previously described using aseptic techniques (63, 64). Spot glucose measurements were also collected by glucose test strips at baseline and periodically throughout the HFD treatment (2 times/wk) to calibrate the telemeters and to determine the accuracy and reliability of the glucose telemeters. Plasma triglycerides and cholesterol were measured in male and female rats on a NFD or HFD using Triglyceride Colorimetric Assay kit (catalog no. 10010303, Cayman Chemical, Ann Arbor, MI) and Cholesterol Fluorometric Assay kit (catalog no. 10007640, Cayman Chemical) per the manufacturer’s protocols.

#### Analytical flow cytometry.

**Fig. 1.**
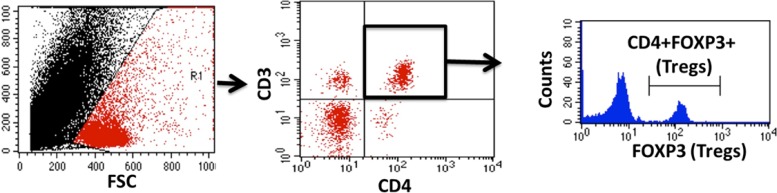
Flow cytometry gating strategy showing the selection of single cells and exclusion of cell debris based on forward scatter (FSC) and side scatter (SSC). T cells were analyzed based on the expression of CD3 and/or CD4. Total CD3^+^ T cells were further gated for expression of RAR-related orphan receptor-γt for T helper 17 cells, and CD3^+^CD4^+^ T cells were further gated for expression of Foxp3 for regulatory T cells (Tregs).

A second set of animals was placed on the HFD or NFD for 4 wk to collect aortas and kidneys for T cell analysis; no additional measurements were made in these animals. Single cell suspensions of thoracic aortas with the adherent fat intact or kidneys were generated as previously described in PBS (*n* = 4–6 male rats and *n* = 6 female rats) (1, 2). Briefly, phenotypic and intracellular analyses were performed by incubating cells with antibodies for T cell surface markers including CD3 (1:100, eBioscience) and CD4 (1:100, BD Biosciences) for 15 min on ice in the dark. After a wash, cells were fixed and permeabilized using fix/perm concentrate (eBioscience, San Diego, CA) before incubation with antibodies for intracellular staining of Foxp3 (1:100, eBioscience) and IL-10 (1:100, Novus) to identify regulatory T cells (Tregs) or RAR-related orphan receptor-γt (1:100, R&D Systems) and IL-17 (1:100, eBioscience) to identify T helper (Th)17 cells. Cells were then washed and run through a four-color flow cytometer (FACS Calibur, BD Biosciences), and data were collected using CellQuest software as previously described (1, 2). An example of the flow cytometry gating strategy used is shown in Fig. 1. T cells were analyzed based on the expression of CD3 and/or CD4. Total CD3^+^ T cells were further gated for expression of RAR-related orphan receptor-γt for Th17 cells, and CD3^+^CD4^+^ T cells were further gated for expression of Foxp3 for Tregs.

#### Statistical analysis.

All data are presented as means ± SE. Flow cytometric data and metabolic cage data were compared using two-way ANOVA. Glucose telemetry, tail cuff, and body weight within each group were analyzed using repeated-measures ANOVA. Between-group measurements were made using two-way ANOVA for tail cuff and body weight and *t*-tests for glucose measurements. Analyses were performed using GraphPad Prism (version 6.0) software (GraphPad Software, La Jolla, CA). For all comparisons, differences were considered statistically significant with *P* < 0.05.

## RESULTS

### 

#### A 4-wk HFD led to greater increases in body weight and total fat intake but not total caloric intake compared with NFD.

Previous studies have shown that male DSS rats exhibit weight gain in response to HFD (47, 48, 62). To determine if there is a sex difference in the impact of HFD on metabolic parameters, male and female DSS rats were placed on a HFD or maintained on a NFD from 12 to 16 wk of age. Although total food intake was less in rats of both sexes on a HFD compared with a NFD at baseline (effect of diet: *P* = 0.0055) and at *week 4* of treatment (effect of diet: *P* < 0.001; Table 1), total fat intake was significantly greater in rats on a HFD compared with NFD in both sexes (effect of diet: *P* < 0.0001; interaction: *P* = 0.13; Table 1). Female rats had greater fat intake than male rats regardless of diet after 4 wk (effect of sex: *P* = 0.035; Table 1). Diet had no effect on total caloric intake (effect of diet: *P* = 0.83; Table 1) but consistent with the greater fat intake in female rats, female rats had greater total caloric intake than male rats regardless of diet after 4 wk (effect of sex: *P* = 0.013; interaction: *P* = 0.52; Table 1).

**Table 1.** Metabolic cage data, including body weight, food intake, total caloric intake, total fat intake, water intake, and urine output, in male and female rats on a NFD or HFD for 4 wk

Metabolic Parameter	Male NFD	Female NFD	Male HFD	Female HFD
Body weight, g				
Baseline	388.8 ± 3.7‡	230.0 ± 4.7	324.0 ± 9.1*‡	220.0 ± 4.1
*Week 4*	459.6 ± 5.8†‡	265.0 ± 11.4†	416.9 ± 5.8*†‡	263.1 ± 4.2†
Food intake, g/day				
Baseline	18.6 ± 0.8	16.8 ± 0.7	14.7 ± 2.8	11.5 ± 0.9
*Week 4*	15.4 ± 1.7†‡	19.2 ± 0.9†	9.8 ± 2.2*†‡	14.3 ± 0.9†
Total caloric intake, kcal/day				
Baseline	72.2 ± 2.9	65.1 ± 2.8	80.2 ± 15	62.8 ± 5.1
*Week 4*	59.7 ± 6.5	53.5 ± 12.2	74.7 ± 3.4	77.8 ± 5.0
Total fat intake, kcal/day				
Baseline	11.9 ± 0.5	10.7 ± 0.5	47.7 ± 9.0*	37.3 ± 3.0*
*Week 4*	9.8 ± 1.1	12.3 ± 0.6	31.8 ± 7.3*	46.3 ± 3.0*†
Water intake, ml/day				
Baseline	29.3 ± 2.7	28.1 ± 2.7	25.1 ± 2.5	18.2 ± 0.8*
*Week 4*	30.0 ± 5.0	37.2 ± 1.9	42.6 ± 6.6	36.7 ± 3.1†
Urine output, ml/day				
Baseline	14.0 ± 1.0	18.4 ± 3.3	11.6 ± 1.1	8.9 ± 1.2*
*Week 4*	18.6 ± 3.2	24.0 ± 2.0	17.1 ± 4.0	13.6 ± 2.0*

All data are expressed as means ± SE; *n* = 5 male rats and 5 female rats. NFD, normal-fat diet; HFD, high-fat diet. Between-group comparisons were made using two-way ANOVA or repeated-measures two-way ANOVA between baseline and final. Within-group comparisons were made using a post hoc Tukey or Sidak multiple-comparisons test, respectively.

* *P* < 0.05 vs. the same-sex NFD-fed control group;

† *P* < 0.05 vs. baseline in the same treatment group in either male or female rats;

‡ *P* < 0.05 vs. female rats of the same treatment group (NFD or HFD).

There was an age-related increase in body weight in both male and female rats from baseline to *week 4* of treatment in each group (Table 1). Body weight in male DSS rats was higher at baseline compared with age-matched female DSS rats and remained significantly higher in male rats at the end of the treatment period regardless of diet (effect of sex: *P* < 0.0001; Table 1). Because of baseline differences in body weight between treatment groups in male rats (Table 1), the percent increase in body weight in response to the HFD or NFD over the 4-wk treatment period was calculated. The HFD resulted in greater increases in body weight compared with the NFD in both male DSS rats (NFD: 18 ± 0.7% increase vs. HFD: 29 ± 2.9% increase) and female DSS rats (NFD: 15 ± 1.4% increase vs. HFD: 20 ± 1.3% increase; effect of diet: *P* = 0.0024). Male rats exhibited greater increases in body weight compared with female rats regardless of diet (effect of sex: *P* = 0.0004); moreover, the increase in body weight with the HFD tended to be greater in male compared with female rats, although this increase did not reach statistical significance (interaction: *P* = 0.09).

Diets high in fat are known to induce hypercholesterolemia and hyperglycemia accompanied by impaired glucose tolerance (52). Terminal plasma triglyceride and cholesterol levels were measured after the 4-wk NFD or HFD treatment. The HFD significantly decreased plasma triglycerides compared with the NFD in male DSS rats (NFD: 137 ± 15 mg/dl vs. HFD: 78 ± 11 mg/dl) but not female DSS rats (NFD: 61 ± 9 mg/dl vs. HFD: 54 ± 9 mg/dl; effect of diet: *P* < 0.0001; effect of sex: *P* = 0.0063; interaction: *P* = 0.023). Plasma cholesterol levels remained unchanged in both male (NFD: 2,698 ± 257 μmol/l vs. HFD: 1,739 ± 403 μmol/l) and female (NFD: 1,736 ± 316 μmol/l vs. HFD: 1,574 ± 315 μmol/l) rats (effect of diet: *P* = 0.104; effect of sex: *P* = 0.10; interaction: *P* = 0.24).

**Fig. 2.**
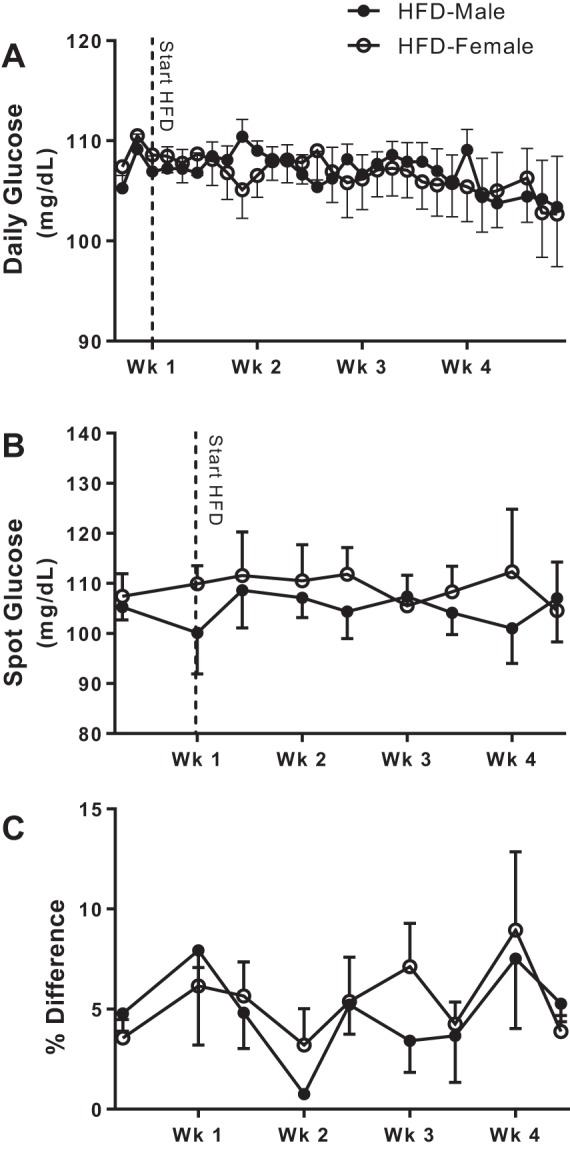
Blood glucose levels were continuously monitored in male (*n* = 5) and female (*n* = 6) Dahl salt-sensitive rats on a high-fat diet (HFD) from baseline until the end of the study (*A*). Spot glucose measurements were also performed periodically over the 4 wk of HFD (*B*), and the percent difference of the spot glucose readings compared with the telemetry readings was calculated to determine the accuracy of the telemetry data (*C*). All data are presented as means ± SE. Glucose telemetry and spot glucose data within each group were analyzed using repeated-measures ANOVA; between-group comparisons were made using *t*-tests. There were no significant differences.

A subset of rats was implanted with telemeters to monitor blood glucose levels throughout the HFD treatment (Fig. 2). Blood glucose levels were not significantly altered by the HFD compared with NFD, and levels were comparable between male and female rats throughout the study, as measured by both glucose telemeters (*P* = 0.33 and *P* = 0.25, repeated-measures ANOVA in male and female rats, respectively; Fig. 2*A*) and spot blood glucose tests (*P* = 0.35 and *P* = 0.48, repeated-measures ANOVA in male and female rats, respectively; Fig. 2*B*). Data from the glucose telemeters were compared with spot glucose measurements to assess the accuracy and reliability of the continuous glucose telemetry readings (Fig. 2*C*). Spot glucose measurements were within 10% of the glucose telemetry data at all time points.

#### Female DSS rats exhibit HFD-induced increases in systolic BP after 4 wk, similar to male rats.

**Fig. 3.**
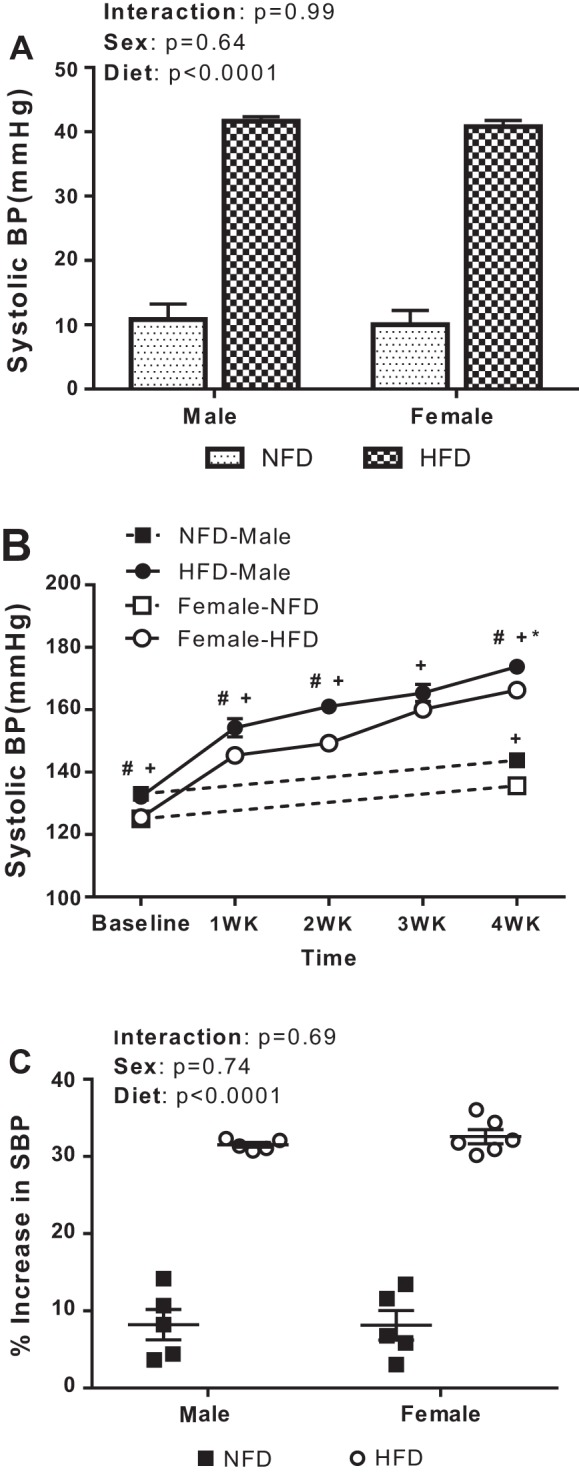
Systolic blood pressure (BP) measured via tail cuff in male (*n* = 5) and female (*n* = 5–6) Dahl salt-sensitive rats expressed as the change (Δ) between baseline and after a 4-wk normal-fat diet (NFD) or high-fat diet (HFD; *A*), expressed as absolute values weekly in the HFD-fed group and at baseline and after 4 wk in the NFD-fed group (*B*) and expressed as the percent increase from baseline (*C*) in both treatment groups. One female rats on the NFD died before final BP measurements. All data are presented as means ± SE. Tail-cuff data within each group were analyzed using repeated-measures ANOVA. Δ and percent increases in systolic BP were compared using two-way ANOVA. +*P* < 0.05 vs. baseline in the same treatment group in both male and female rats; *P < 0.05 vs. same-sex NFD-fed control group; #*P* < 0.05 vs. female rats of same treatment group (NFD or HFD).

In many genetic and diet-induced models of hypertension, male rats exhibit greater BP increases than female rats (33, 65). To determine if there is a sex difference in HFD-induced increases in BP in DSS rats, systolic BP was measured at baseline and at the end of the study in rats on a NFD and weekly in male and female DSS rats on a HFD. A 4-wk HFD resulted in significant increases in systolic BP in both male and female DSS rats compared with NFD (effect of diet: *P* < 0.0001; Fig. 3, *A* and *B*), yet BP was higher in male compared with female rats throughout the study (effect of sex: *P* < 0.0001; Fig. 3*B*). Although BP increased in all rats over the course of the 4-wk treatment period, HFD led to greater percent increases in systolic BP in both male and female rats compared with NFD, and the increase was comparable between the sexes (effect of diet: *P* < 0.0001; effect of sex: *P* = 0.74; interaction: *P* = 0.69; Fig. 3*C*).

#### Both male and female DSS rats exhibit an increase in the percentage of CD4^+^ T cells and a proinflammatory T cell phenotype in the aorta with perivascular adipose tissue after a HFD.

**Fig. 4.**
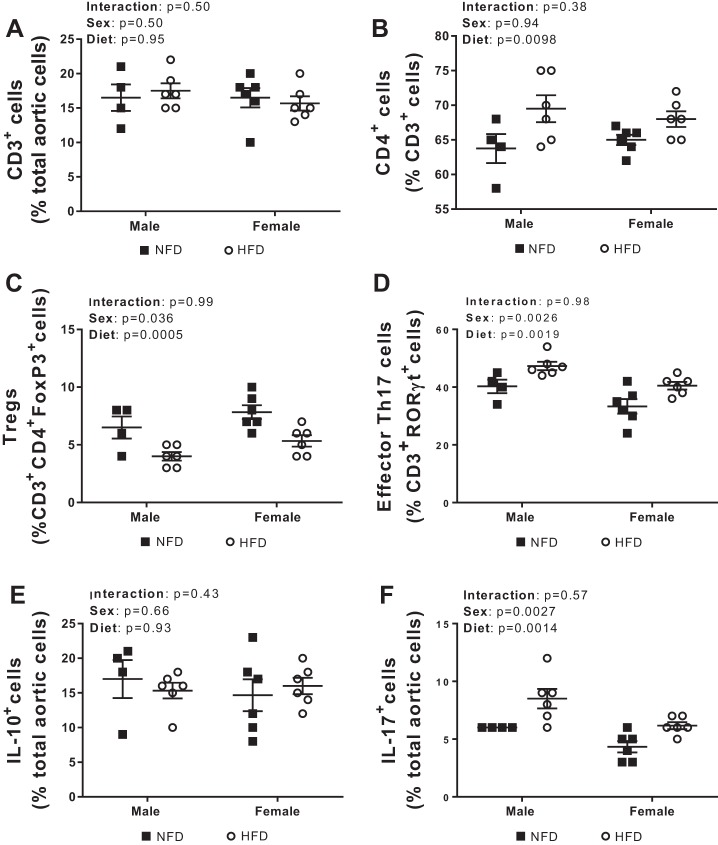
T cell profile in aortas with adherent fat intact of 16-wk-old male (*n* = 4–6) and female (*n* = 6) Dahl salt-sensitive rats treated with a control normal-fat diet (NFD) or high-fat diet (HFD). Shown are percentages of total T cells (*A*), CD4^+^ T cells (*B*), Foxp3^+^ T regulatory cells (Tregs; *C*), and related orphan receptor (ROR)γt^+^ T helper (Th)17 cells (*D*), IL-17^+^ cells (*E*), and IL-10^+^ cells (*F*). All data are expressed as means ± SE. Flow cytometric data were compared using two-way ANOVA.

The onset of hypertension is accompanied by the accumulation of T cells and inflammatory mediators in the vasculature, specifically in the adventitia and perivascular fat (20, 29, 74). To determine if HFD-induced increases in BP are accompanied by changes in the T cell profile in the vasculature, flow cytometric analysis was performed on the thoracic aorta with the adherent fat intact. Although a 4-wk HFD had no effect on the percentage of total CD3^+^ T cells (effect of diet: *P* = 0.95; interaction: *P* = 0.50; Fig. 4*A*), the HFD increased the percentage of CD4^+^ T cells in the aorta compared with the NFD regardless of sex (effect of diet: *P* = 0.0098; interaction: *P* = 0.38; Fig. 4*B*). Moreover, the percentage of total T cells and CD4^+^ T cells in the aorta was comparable between the sexes (total T cells, effect of sex: *P* = 0.50; CD4^+^ T cells, effect of sex: *P* = 0.94).

A role for CD4^+^ T cell subsets, specifically Tregs and Th17 cells and their respective cytokines, have been demonstrated in the pathogenesis of hypertension (37, 42, 66, 73). The HFD decreased the percentage of anti-inflammatory Tregs (effect of diet: *P* = 0.036; interaction: *P* = 0.68; Fig. 4*C*) and increased the percentage of Th17 cells (effect of diet: *P* = 0.0019; interaction: *P* = 0.98; Fig. 4*D*) as well as IL-17^+^ cells (effect of diet: *P* = 0.0019; interaction: *P* = 0.99; Fig. 4*F*) regardless of sex. The HFD had no effect on the percentage of IL-10^+^ cells regardless of sex (effect of diet: *P* = 0.93; interaction: *P* = 0.43; Fig. 4*E*). Although female rats had a higher percentage of Tregs than male rats (effect of sex: *P* = 0.0005; Fig. 4*C*), the percentage of aortic cells that expressed the anti-inflammatory cytokine IL-10 were similar between the sexes (effect of sex: *P* = 0.66; Fig. 4*E*). In contrast, male rats had a higher percentage of Th17 cells than female rats (effect of sex: *P* = 0.0026; Fig. 4*B*); consistent with the finding, a higher percentage of aortic cells expressed the proinflammatory cytokine IL-17 (effect of sex: *P* = 0.0014; Fig. 4*F*).

#### Male DSS rats exhibit greater HFD-induced increases in the percentage of proinflammatory T cell infiltration in the kidney than female rats.

**Fig. 5.**
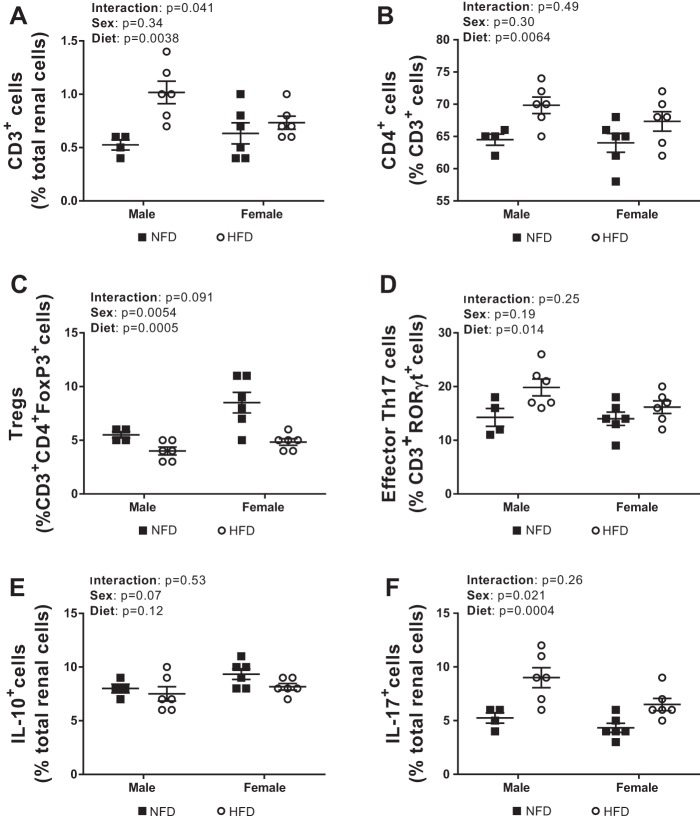
T cell profile in kidneys of 16-wk-old male (*n* = 4–6) and female (*n* = 6) Dahl salt-sensitive rats treated with a control normal-fat diet (NFD) or high-fat diet (HFD). Shown are percentages of total T cells (*A*), CD4^+^ T cells (*B*), Foxp3^+^ T regulatory cells (Tregs; *C*), and related orphan receptor (ROR)γt^+^ T helper (Th)17 cells (*D*), IL-17^+^ cells (*E*), and IL-10^+^ cells (*F*). All data are expressed as means ± SE. Flow cytometric data were compared using two-way ANOVA.

The kidney plays an important role in the long-term regulation of BP, and T cell infiltration in the kidney has been shown to impact BP in male experimental models (28). To determine the impact of sex and diet on renal T cell infiltration, analytical flow cytometry was used to measure the percentage of total T cells in male and female DSS rats fed a NFD or HFD. A 4-wk HFD led to an increased percentage of total renal CD3^+^ T cells compared with a NFD in both sexes, although the increase was greater in male rats (effect of diet: *P* = 0.0038; effect of sex: *P* = 0.34; interaction: *P* = 0.041; Fig. 5*A*). The HFD also significantly increased the percentage of CD4^+^ T cells in both male and female rats, and the increase was comparable between the sexes (effect of diet: *P* = 0.0064; effect of sex: *P* = 0.30; interaction: *P* = 0.49; Fig. 5*B*).

The HFD also led to a decrease in the percentage of immunosuppressive Tregs (effect of diet: *P* = 0.0005; interaction: *P* = 0.091; Fig. 5*C*) and an increase in the percentage of proinflammatory Th17 cells regardless of sex compared with rats maintained on a NFD (effect of diet: *P* = 0.014; interaction: *P* = 0.25; Fig. 5*D*). The decrease in the percentage of Tregs was not accompanied by any changes in IL-10^+^ cells in response to HFD in either sex (effect of diet: *P* = 0.12; interaction: *P* = 0.11; Fig. 5*E*). However, the HFD did increase the percentage of IL-17^+^ cells compared with the NFD regardless of sex (effect of diet: *P* = 0.0004; interaction: *P* = 0.26, Fig. 5*F*). Female DSS rats had a greater percentage of immunosuppressive Tregs than male DSS rats (effect of sex: *P* = 0.0054; Fig. 5*C*) and tended to have more IL-10^+^ cells, although this did not reach statistical significance (effect of sex: *P* = 0.07; Fig. 5*E*). Although the percentage of proinflammatory Th17 cells was comparable between the sexes (effect of sex: *P* = 0.19; Fig. 5*D*), male DSS rats had a greater percentage of IL-17^+^ cells than female DSS rats (effect of sex: *P* = 0.021; Fig. 5*F*).

## DISCUSSION

The primary novel finding of the present study is that a 4-wk HFD results in a similar increase in systolic BP in male and female DSS rats. HFD-induced increases in BP were associated with an increased percentage of total T cells in the kidney as well as an increased percentage of renal and aortic proinflammatory Th17 cells; however, male rats had a greater percentage of renal Th17 cells compared with female rats. The HFD also decreased the percentage of aortic Tregs, and female rats had a greater percentage of Tregs than male rats regardless of diet. These data suggest that a 4-wk HFD has a main effect on immune cell activation that is not sex specific. Based on the finding that the increase in BP is comparable between the sexes despite differences in aortic and renal T cell profiles, the mechanisms by which a HFD impacts BP may be distinct between male and female rats. Future studies will assess the relative contribution of proinflammatory Th17 cells and anti-inflammatory Tregs to HFD-induced hypertension in male and female DSS rats.

Fat-enriched diets are commonly used to mimic symptoms of metabolic syndrome, including increased adiposity, hypertension, dyslipidemia, and impaired glucose tolerance in rodents (7, 8). However, the responses in these metabolic parameters can vary widely depending on the rat strain and sex as well as the source of dietary fat (38). In the present study, we found that a 4-wk lard-enriched HFD led to a greater percent increase in body weight relative to the control NFD in both male and female DSS rats, although this increase tended to be higher in male DSS rats. Although the HFD protocol did result in an increase in body weight relative to the control NFD in male rats, the increase was relatively mild. However, based on the knowledge that visceral fat as opposed to subcutaneous fat increases cardiovascular disease risk (34), an alteration in the distribution of body fat could have a significant impact on cardiovascular outcomes. Moreover, it is possible that reduced energy expenditure contributes to increases in body weight in male DSS rats since caloric intake remained unchanged with HFD (15).

HFD-induced increases in body weight were accompanied by lower levels of plasma triglycerides in male but not female DSS rats with no change in plasma cholesterol levels in either sex. These data are consistent with other studies with respect to male DSS rats maintained on a HFD (48, 62), suggesting that the DSS strain is resistant to HFD-induced increases in plasma lipids. The mechanism by which DSS rats handle lipids to allow for a reduction in triglycerides and no change in cholesterol has not been examined in either sex. There are, however, a handful of studies that have reported a decrease in plasma triglycerides with a HFD in male rats, suggesting that this finding may not be unique to DSS rats (62). Male B6D21 mice exhibit decreases in plasma triglycerides after 3 wk of high-fat feeding because of improved triglyceride clearance from the blood (53). Similarly, male Wistar and Sprague-Dawley rats fed a HFD containing 15% unsaturated fat from corn oil or 20% lard, respectively, also show reduced plasma triglycerides, which was thought to be because of an increase in hepatic triglyceride storage coupled with reduced very low-density lipoprotein secretion (17, 27). Blood glucose levels were also maintained on a HFD in DSS rats. To the best of our knowledge, there is no previous evidence showing that DSS rats maintain blood glucose in response to a HFD, although plasma blood glucose levels increase after 8 wk of high-fat feeding in male DSS rats (48). Thus, it is possible that an extended HFD treatment would impact both lipid and glucose handling. It is important to note, however, that phenotypic differences in BP responses to dietary factors in DSS rats have been reported based on vendor (77) and metabolic and cardiovascular responses to fat-enriched diets can vary widely depending on dietary fat source and content. Therefore, it is important to note all of these factors when drawing conclusions. In contrast to our study, the male DSS rats exhibiting an increase in blood glucose with 8 wk of high-fat feeding were purchased from Japlan and maintained on Research Diets (New Brunswick, NJ) high-fat pellets (45% kcal from fat) (48). Although the primary source of fat is the same (lard), the caloric density was greater compared with the HFD from Bio-Serv (60% kcal from fat) previously used (62), and in the present study, DSS rats were maintained in different colonies, which influenced metabolic responses.

We found that male DSS rats exhibit an increase in systolic BP when maintained on a normal-salt HFD, as previously reported (36, 48, 62), and we further extend these findings to female rats. It should also be noted that there was an age-related increase in systolic BP in rats maintained on the NFD over the course of the 4-wk study in both male and female DSS rats. Although absolute BP measurements were lower in female than male DSS rats on both the NFD and HFD, both sexes exhibited a similar increase in BP in response to a 4-wk HFD, suggesting that female DSS rats are not resistant to HFD-induced hypertension. This finding is in contrast to what has been shown in other studies that have examined sex differences in diet-induced hypertension specifically in rat models (16, 57, 60, 65). Male Wistar rats exhibit significant increases in systolic BP in response to either a high-fructose diet or HFD compared with control rats, whereas BP did not change from that of controls in female rats (16, 65) even when treated for an extended period of time. Male Fischer rats fed a HFD also exhibit increases in BP after 2 mo of treatment, whereas BP does not begin to increase in female Fischer until 12 mo of high-fat feeding (57). Similarly, female Sprague-Dawley rats remain normotensive compared with male Sprague-Dawley after a 10-wk cafeteria diet (10). However, DSS rats may be unique in that they are prone to develop diet-induced hypertension. Both male and female DSS rats develop hypertension in response to a high-salt (8% NaCl) diet, although the magnitude of the BP increase is greater in male DSS rats (>30 mmHg) than in female DSS rats (>10 mmHg) compared with rats maintained on a low-salt (0.15% NaCl) diet (24). Therefore, the finding that male and female DSS rats develop a similar degree of hypertension on a HFD is intriguing and may be consistent with clinical literature where women are more at risk for cardiovascular disease in response to dietary fat (35, 51).

The mechanism mediating the greater susceptibility of female DSS rats to develop HFD-induced hypertension compared with salt-induced increases in BP is unknown. Intrinsic differences may confer greater susceptibility to HFD-induced hypertension in female DSS rats compared with other strains that is also not seen with other types of diet. Several quantitative trait loci have been identified in DSS rats that confer hypertension (14); however, whether there is a sex difference in the expression of these genes and to different types of hypertensive stimuli, including a HFD, have not been determined experimentally. For example, it has been shown that elevated levels of BTG antiproliferation factor 2, which is highly expressed in kidney proximal tubules, before salt challenge predisposes female SS13BN rats to hypertension (26). Therefore, it is possible that certain genetic loci promote HFD-induced increases in BP in female DSS rats to such an extent that the sex difference observed in other strains or in response to other hypertensive stimuli, such as high salt, is eliminated. Alternatively, high-fat feeding has been shown to reduce expression of estrogen receptor α in adipose tissue (18), and it has been postulated that the competitive binding of circulating oxysterols, such as 27-hydroxycholesterol, to estrogen receptor α can block the beneficial effects of estrogen in the vasculature in response to an HFD (72). This is an intriguing hypothesis because female sex hormones have been shown to protect against high salt-induced increases in BP in female DSS rats leading to the loss of the sex difference in the magnitude of the BP response (5, 24, 25). Future studies are needed to address the role of estrogen in HFD-induced hypertension in female DSS rats.

The rapid BP response to a HFD, especially under a relatively short treatment period, appears to be unique to DSS rats and may be unique to DSS rats from Envigo maintained on the Bio-Serv HFD. Fifteen weeks of high-fat feeding (45% fat from lipids and 17% of energy from sucrose, D12451, Research Diets) had a negligible effect on systolic BP in male Sprague-Dawley and Wistar rats (purchased from Harlan Laboratories, Santiga, Spain) compared with standard chow diet (43). If the HFD treatment (D12451, Research Diets) is extended to 32 wk; however, male Sprague-Dawley rats (purchased from Harlan, Indianapolis, IN) exhibit a 20-mmHg increase in mean arterial pressure (11). In contrast, in SHRs, a HFD (45% kcal from fat) reduced BP in male rats (61). Although in a separate study male SHRs exhibited significant increases in systolic BP after 15 wk of HFD (60% kcal from fat, 211 ± 9 mmHg) compared with control diet (175 ± 11 mmHg) (9), only the final values were reported; therefore, there is no indication when BP started to increase in these animals.

The mechanism underlying HFD-induced hypertension in DSS rats is unknown, but recent studies have implicated the immune system and T cells. Specifically, the use of immunosuppressive agents or genetic deletion of components of the immune system in several pioneering studies have demonstrated a role for immune cells in the development of diet-induced hypertension and kidney damage in male DSS rats (12, 13, 44–46, 58, 62). Treatment of male DSS rats with the lymphocyte inhibitor MMF or genetic deletion of recombinase activating gene 1 attenuates salt-sensitive hypertension and immune cell infiltration in the kidney (45, 46). Of the immune cells infiltrating the kidney, T cells were found to represent the largest fraction (12). Furthermore, genetic deletion of CD247, which is required for T cell activation and proliferation, reproduces the same BP-lowering effects as the immunosuppressant MMF or recombinase activating gene 1 deletion, strongly suggesting that dysregulation in the T cell response underlies salt-sensitive hypertension in male DSS rats (58). Recent studies have also shown that increases in BP in male DSS rats in response to a HFD are attenuated by MMF, and this corresponds to a decrease in renal inflammation (62). In the present study, we examined T cells in both the aorta with the perivascular adipose tissue intact and the kidney. The accumulation of T cells and the associated inflammatory mediators in the vasculature can reduce nitric oxide bioavailability and promote vascular remodeling to contribute to hypertension development (50). In the kidney, inflammation can disrupt the expression and activity of Na^+^ transporters along the nephron leading to impaired pressure natriuresis as well as Na^+^ and water retention leading to elevations in BP (50).

Although there are limited data on T cell infiltration in the vasculature of DSS rats, it has been shown that male DSS rats exhibit increased expression of inflammatory markers and mediators as well as aortic CD8^+^ T cell infiltration compared with normotensive SSBN2 control rats (73). Furthermore, angiotensin II-induced hypertension leads to aortic accumulation of T cells particularly in the adventitia and perivascular fat of male C57BL/6 mice (20, 29, 74). Consistent with these data, in the present study, male DSS rats exhibited a significant increase in systolic BP when placed on a normal-salt HFD for 4 wk, and this was accompanied by enhanced percentages of renal total T cells, aortic CD4^+^ T cells, and Th17 cells in both tissues.

We found that a 4-wk HFD also increased the percentages of aortic CD4^+^ T cells in female DSS rats. Although there was no change in the percentage of renal CD4^+^ T cell infiltration in response to a HFD in either sex, there were HFD- and sex-specific effects on CD4^+^ T cell subtypes. Numerous studies have implicated Tregs and Th17 cells and their respective cytokines in BP control (3, 4, 30, 31, 37, 42, 66, 73), where Th17 cells and IL-17 cytokines are prohypertensive and Tregs and IL-10 cytokines are antihypertensive. Previous studies by our group have shown that hypertensive female animals have more Tregs than male animals because of a BP-dependent increase in Tregs only in female animals (67, 69, 76). In contrast with previous studies by our group and others (19, 29, 56, 67, 69), male and female animals exhibited similar increases in the percentage of Th17 cells in response to a HFD. In addition, although a 4-wk HFD increased the percentage of Th17 cells in both sexes, male animals maintained higher percentages of Th17 cells in the aorta and IL-17^+^ cells in both the aorta and kidney. Future studies will directly test the hypothesis that T cells contribute to the increase in BP with a HFD. Future studies will also assess the impact of a HFD on additional immune cells, including macrophages. HFD also promotes the infiltration of monocytes and macrophages (40), and a role for the innate immune system in hypertension has been previously identified (50). Future studies will also measure CD8^+^ T cells, which have also been implicated in hypertension (36, 38, 71). Interestingly, male mice fed a HFD exhibit greater increases in CD8^+^ T cells compared with CD4^+^ T cells in adipose tissue (47), and we have previously shown that female SHRs have more CD8^+^ T cells in the kidney compared with male SHRs (69). Furthermore, sympathetic hyperactivity has been linked to HFD-induced increases in BP (32) and T cell activation in the kidney that underlie angiotensin II-induced hypertension in C57BL/6 mice (75). Given that there are well-documented sex differences in sympathetic nervous system activity that can impact BP control (6, 23), it would be of additional interest to examine the role of increased sympathetic outflow in HFD-induced hypertension and immune cell activation in both male and female DSS rats.

In contrast to what was observed with proinflammatory T cells, the HFD decreased the percentages of aortic and renal Tregs, although female rats had a greater percentage of Tregs than male rats in both the kidney and aorta regardless of diet. This is in contrast to other studies that showed a compensatory increase in Tregs in female animals in response to hypertensive stimuli that was not observed in male animals (54, 67, 69, 76). This raises the possibility of a dysfunction in the Treg response to HFD in female DSS rats that may contribute to the susceptibility to HFD-induced increases in BP. The adoptive transfer of Tregs has been shown to blunt aldosterone-induced hypertension (30) as well as angiotensin II-induced hypertension in C57BL/6 mice (3, 37), highlighting an important antihypertensive role of this T cell subset (3). Therefore, future studies are required to determine if *1*) Tregs attenuate HFD-induced increases in BP in both male and female DSS rats and *2*) there is a sex difference in the antihypertensive capacity of Tregs in response to a HFD.

There are limitations in the present study. First, BP was measured by tail cuff as opposed to telemetry. Second, body fat distribution was not assessed. It would be of interest to determine whether there is a redistribution of body fat, specifically an accumulation of visceral fat in response to the HFD in male and female DSS rats. In addition, the difference in baseline body weight in male DSS rats on NFD versus HFD raises the following question: would the same results be obtained if the rats with a higher body weight were placed on the HFD? Although the only definitive way to address this question would be to repeat the entire study, because the HFD led to greater percent increases in BP in both sexes compared with the NFD, and only the male rats exhibited the baseline difference in body weight, the data suggest that the diet itself as opposed to body weight drives the increase in BP. Third, vascular T cells were measured in a conduit artery versus a resistance vascular bed. Finally, immune assessments were only performed after BP was markedly elevated by HFD, not allowing for a determination as to whether immune cell changes precede hypertension development. However, it has been recently reported that increased pressure promotes the initial infiltration of T cells into the kidney of male DSS rats (14).

## GRANTS

This work was funded by National Heart, Lung, and Blood Institute Grants R0-HL-127091 and P01-HL-134604 (to J. Sullivan) and American Heart Association Grant 17EIA33410565 (to J. Sullivan).

## DISCLOSURES

No conflicts of interest, financial or otherwise, are declared by the authors.

## AUTHOR CONTRIBUTIONS

L.E.T. and J.C.S. conceived and designed research; L.E.T., E.E.G., and J.B.M. performed experiments; L.E.T., E.E.G., B.B., and J.C.S. analyzed data; L.E.T., E.E.G., J.B.M., B.B., and J.C.S. interpreted results of experiments; L.E.T., E.E.G., and J.C.S. prepared figures; L.E.T. and J.C.S. drafted manuscript; L.E.T., E.E.G., J.B.M., B.B., and J.C.S. edited and revised manuscript; L.E.T., E.E.G., J.B.M., B.B., and J.C.S. approved final version of manuscript.
